# AMTAC-19, a Spiro-Acridine Compound, Induces In Vitro Antitumor Effect via the ROS-ERK/JNK Signaling Pathway

**DOI:** 10.3390/molecules29225344

**Published:** 2024-11-13

**Authors:** Valgrícia Matias de Sousa, Sâmia Sousa Duarte, Rafael Carlos Ferreira, Natália Ferreira de Sousa, Marcus Tullius Scotti, Luciana Scotti, Marcelo Sobral da Silva, Josean Fechine Tavares, Ricardo Olímpio de Moura, Juan Carlos Ramos Gonçalves, Marianna Vieira Sobral

**Affiliations:** 1 Postgraduate Program in Natural Products and Bioactive Synthetics, Federal University of Paraíba, João Pessoa 58051-970, PB, Brazil; 2 Drug Development and Synthesis Laboratory, Department of Pharmacy, State University of Paraíba, João Pessoa 58070-450, PB, Brazil

**Keywords:** colorectal carcinoma, oxidative stress, MAPKs

## Abstract

Colorectal cancer remains a significant cause of mortality worldwide. A spiro-acridine derivative, (*E*)-1′-((4-bromobenzylidene)amino)-5′-oxo-1′,5′-dihydro-10*H*-spiro[acridine-9,2′-pyrrole]-4′-carbonitrile (AMTAC-19), showed significant cytotoxicity in HCT-116 colorectal carcinoma cells (half maximal inhibitory concentration, IC50 = 10.35 ± 1.66 µM) and antioxidant effects after 48 h of treatment. In this study, Molegro Virtual Docker v.6.0.1 software was used to investigate the interactions between AMTAC-19 and the Extracellular Signal-Regulated Kinase 1 (ERK1), c-Jun N-terminal Kinase 1 (JNK1), and p38 Mitogen-Activated Protein Kinase α (p38α MAPK). In vitro assays were conducted in HCT-116 cells to evaluate the effect of AMTAC-19 on the modulation of these proteins’ activities using flow cytometry. Furthermore, the 3-(4,5-dimethylthiazol-2-yl)-2,5-diphenyltetrazolium bromide (MTT) assay in the presence or absence of ERK1/2, JNK, and p38 MAPK inhibitors was used to evaluate the involvement of these enzymes in AMTAC-19 cytotoxicity. ROS production was assessed using the 2,7-dichlorodihydrofluorescein diacetate (DCFH-DA) assay at various incubation times (30 min, 1 h, 6 h, 12 h, and 24 h), and the MTT assay using N-acetyl-L-cysteine (NAC) was performed. In silico results indicated that AMTAC-19 interacts with ERK1, JNK1, and p38α MAPK. Additionally, AMTAC-19 activated ERK1/2 and JNK1 in HCT-116 cells, and its cytotoxicity was significantly reduced in the presence of ERK1/2 and JNK inhibitors. AMTAC-19 also induced a significant increase in ROS production (30 min and 1 h), while NAC pretreatment reduced its cytotoxicity. These findings support AMTAC-19′s in vitro antitumor effect through ROS-dependent activation of ERK and JNK pathways.

## 1. Introduction

**Figure 1:**
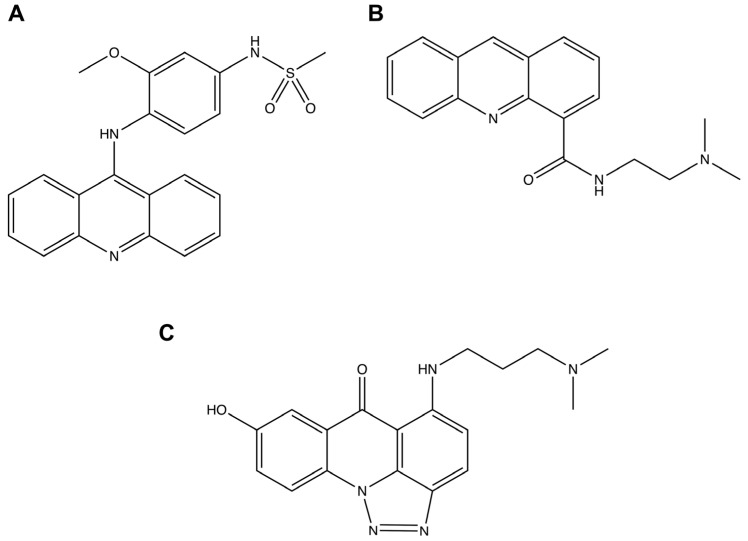
Chemical structures of acridine-based derivatives in clinical trials. (**A**) amsacrine (m-AMSA); (**B**) *N*-[2-(dimethylamino)ethyl]acridine-4-carboxamide (DACA); and (**C**) triazoloacridone.

Colorectal cancer (CRC) has a high mortality rate, with 0.9 million deaths in 2020 [1]. Over the next two decades, a 70% increase in CRC mortality is expected [2], highlighting the need for advancements in treatment and the development of new drugs. In this context, acridine compounds, composed of two benzene rings and one pyridine ring [3], represent a class of heterocyclic molecules with potential for chemical modifications to develop new anticancer drug candidates [4,5,6,7,8], including anti-CRC effects [9,10,11,12,13,14,15,16,17]. Currently, several acridine-based compounds are undergoing clinical trials for cancer treatment, such as amsacrine (m-AMSA), *N*-[2-(dimethylamino)ethyl]acridine-4-carboxamide (DACA), and triazoloacridone [18] (Figure 1).

The antitumor activity of acridine derivatives has been attributed to the planarity of their structure, allowing them to intercalate between DNA base pairs, as well as inhibiting topoisomerase I and II enzymes [3,17,19,20,21,22,23,24]. Additionally, the cytotoxicity of these molecules involves the induction of apoptosis [4,11,25,26,27] and oxidative stress [10,12,28,29,30] and modulation of Mitogen-Activated Protein Kinases (MAPKs) signaling pathways [31,32,33,34,35].

Reactive Oxygen Species (ROS) are small molecules derived from molecular oxygen [36] and are involved in various biological processes. In cancer, basal ROS levels are significantly elevated, contributing to cellular events such as angiogenesis [37]. However, it has been demonstrated that further increases in intracellular ROS levels may be associated with the activation of apoptosis following the modulation of intracellular signaling pathways, including MAPKs [38].

MAPKs consist of three subfamilies: extracellular signal-regulated kinase (ERK), c-Jun N-terminal kinase (JNK), and p38 kinase (p38 MAPK) [39]. These proteins regulate various cellular events following the phosphorylation of their targets [40,41]. In cancer, the MAPK signaling pathway has been associated with tumor development and progression, including CRC [42,43]. However, ROS-mediated activation of ERK1/2, JNK, and p38 MAPK has been linked to an anti-CRC effect [42,43,44].

The antitumor activity of a new class of acridine molecules, spiro-acridine compounds, has been demonstrated. These structures are obtained through cyclization reactions, producing a five- or six-membered spiro ring attached to the C9 carbon of acridine [45]. In an Ehrlich ascitic carcinoma model, the spiro-acridine compounds AMTAC-17 and AMTAC-06 reduced tumor growth through antiangiogenic and immunomodulatory actions [46,47]. Additionally, AMTAC-06 induced cytotoxicity in the HCT-116 colorectal carcinoma cell line by triggering apoptosis and exhibiting antioxidant activity [10].

**Figure 2:**
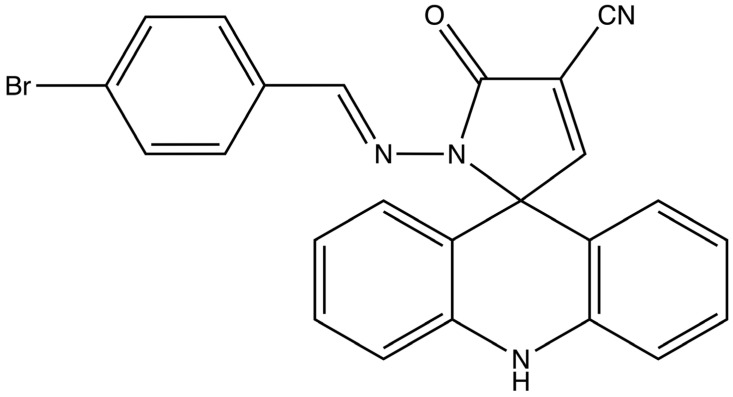
Chemical structure of (*E*)-1′-((4-bromobenzylidene)amino)-5′-oxo-1′,5′-dihydro-10*H*-spiro[acridine-9,2′-pyrrole]-4′-carbonitrile (AMTAC-19).

More recently, the cytotoxicity of a new spiro-acridine compound, (*E*)-1′-((4-bromobenzylidene)amino)-5′-oxo-1′,5′-dihydro-10*H*-spiro[acridine-9,2′-pyrrole]-4′-carbonitrile (AMTAC-19) (Figure 2), has been demonstrated in the HCT-116 cell line. This effect involves cell cycle arrest, apoptosis induction, and antioxidant effects after 48 h of treatment [48].

AMTAC-19 has AMTAC-1 as its prototype and was obtained from 2-cyano-N’-(4-bromo-benzylidene)-acetohydrazide and 9-carboxyaldehyde-acridine, followed by spontaneous cyclization (Figure S1). AMTAC-19 features a bromine (Br) atom in the para position of the phenyl ring and is a stable compound that does not undergo hydrolysis in an aqueous environment or experience interference from direct light during the experimental period. This stability can be attributed to its aromatic system, as observed for other spiro-acridine compounds [3,23,47]. AMTAC-19 and its structural congener AMTAC-06, which contains a chlorine (Cl) atom in the para position of the phenyl ring (Figure S2), exhibit similar cytotoxicity against HCT-116 cell lines, with IC50 values of 10.35 and 12.62 μM, respectively [10,48].

The Br and Cl atoms have similar electronic, lipophilic, and steric properties. Halogen substitution in the para position of the phenyl ring has proven essential for the in vitro activity of the new spiro-acridine derivatives, favoring affinity for targets such as topoisomerase II and DNA [3]. Thus, the synthesis of AMTAC-19 and the investigation of its anti-CRC effects expand the panel of molecules with pharmacological potential for the treatment of this disease.

On the other hand, the antitumor assays for AMTAC-17, which presents methoxy groups in the meta and para positions (Figure S2), were conducted exclusively in an in vivo model (Ehrlich ascitic carcinoma—murine mammary carcinoma) [47], which makes it difficult to compare its effects with the results obtained from the in vitro investigation of the antitumor activity of AMTAC-06 and AMTAC-19 in HCT-116 cells.

Therefore, considering previous data showing the apoptotic effect of AMTAC-19 on the HCT-116 cell line and literature data on the antitumor role of MAPKs in CRC, we present here the molecular docking prediction between AMTAC-19 and ERK1, JNK1, and p38α MAPK enzymes, as well as the in vitro effect of AMTAC-19 treatment on the modulation of these proteins and the initial redox state in human colorectal cancer cells HCT-116.

## 2. Results

### 2.1. AMTAC-19 Exhibits Favorable Interaction with Extracellular Signal-Regulated Kinase 1 (ERK1), c-Jun N-Terminal Kinase 1 (JNK1), and p38 Mitogen-Activated Protein Kinase α (p38α MAPK) in Molecular Prediction

The molecular docking predictions between AMTAC-19 or Protein Data Bank (PDB) ligands (pyridine carbamate inhibitor, pyrazoloquinolone inhibitor, and doramapimod—BIRB-796) and Extracellular Signal-Regulated Kinase 1 (ERK1; PDB: 5LCJ), c-Jun N-terminal Kinase 1 (JNK1; PDB: 2G01), and p38 Mitogen-Activated Protein Kinase α (p38α MAPK; PDB ID: 1R39) were generated using the Moldock score (kJ·mol^−1^). More negative values indicated a higher affinity for the targets. The interaction probability value (*p*) was generated from the standardization of compound values based on the binding energy values of the molecule with the lowest energy.

The RMSD value was 0.9715 for the co-crystallized pyridine carbamate ligand of ERK 1/2 and 0.1129 for the pyrazoloquinolone ligand of JNK1 protein, indicating that the generated poses correctly positioned the ligand in the active site and that the program provided satisfactory values for docking validation.

Table 1 shows the binding energy values and interaction probabilities of the AMTAC-19 compounds, PDB ligands, and the p38α MAPK ligand doramapimod (BIRB-796).

AMTAC-19 showed probability values for ERK1 (−84.735), JNK1 (−74.978), and p38α MAPK (−107.433), indicating favorable interactions between this compound and its targets.

**Figure 3:**
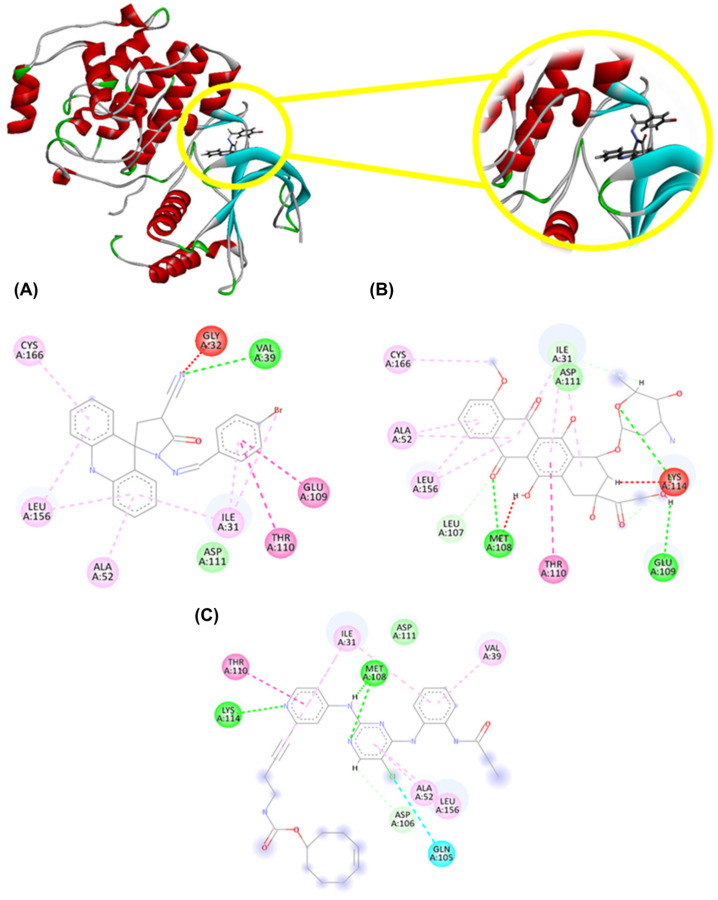
2D and 3D interactions between (**A**) AMTAC-19, (**B**) doxorubicin (DXR), (**C**) PDB Ligand (pyridine carbamate inhibitor), and ERK1 (PDB: 5LCJ). Legend: pink and blue: hydrophobic interactions; red: steric interactions; green: hydrogen bond interactions; CYS: cysteine; GLY: glycine; VAL: valine; LEU: leucine; ALA: alanine; ASP: aspartic acid; ILE: isoleucine; THR: threonine; GLU: glutamic acid; MET: methionine; and GLN: glutamine.

As observed in Figure 3, AMTAC-19 interacts with the active site active site corresponding to the N-Terminal domain referring to the ATP (Adenosine Triphosphate) binding region of ERK1 through hydrophobic interactions (dashed lines in pink and blue), steric interactions (dashed lines in red), and hydrogen bonds (dashed line in green). The hydrophobic interactions corresponded to the alkyl, π-alkyl, and π-π stacked types and were primarily observed in the aromatic rings of the structure and at the bromine (Br) site through the residues Cys166 (1 interaction), Leu156 (2 interactions), Ala52 (1 interaction), Ile31 (3 interactions), Thr110 (1 interaction), and Glu109 (1 interaction). Hydrogen bond interactions were observed at the nitrile group and other nitrogen (N) atoms in the structure through the residues Asp111 (1 interaction) and Val39 (1 interaction). Additionally, a non-favorable interaction was noted at the nitrile group through the residue Gly32. AMTAC-19 exhibited interactions like those established by the compounds doxorubicin (DXR) and the PDB ligand (hydrophobic interactions: Leu156, Ala52, Ile31, and Thr110; hydrogen bond: Asp111).

**Figure 4:**
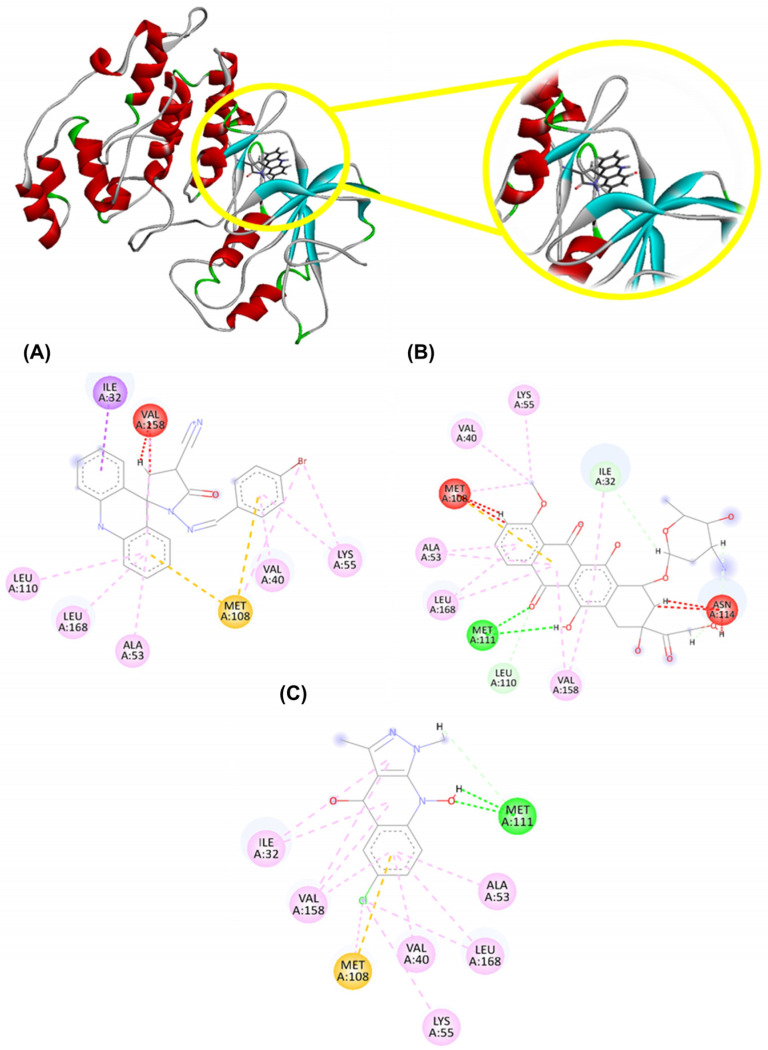
2D and 3D interactions between (**A**) AMTAC-19, (**B**) doxorubicin (DXR), (**C**) PDB Ligand (pyrazoloquinolone inhibitor) and JNK1 (PDB: 2G01). Legend: pink and blue: hydrophobic interactions; red and orange: steric interactions; green: hydrogen bond interactions; LYS: lysine; ASN: asparagine; VAL: valine; LEU: leucine; ALA: alanine; ILE: isoleucine; and MET: methionine.

The molecular interaction between AMTAC-19 and JNK1 involved hydrophobic interactions corresponding to the alkyl, π-alkyl, and π-σ types, related to interactions in the aromatic rings of the structure and at the bromine (Br) site, through the residues Ile32 (1 interaction), Val158 (1 interaction), Leu110 (1 interaction), Leu168 (1 interaction), Ala53 (1 interaction), Met108 (1 interaction), Val40 (1 interaction), and Lys55 (2 interactions). In addition, steric interactions were also observed between AMTAC-19 and the JNK1 active site (unfavorable interactions (dashed lines in red) and π-anion interactions (dashed line in orange)), through the residues Met108 (2 interactions) and Val158 (1 interaction). These interactions were visualized in the alpha domain of the site related to ATP (Adenosine Triphosphate, which corresponds to the active site region of the enzyme, this region being close to the allosteric pocket (Liu et al., 2006 [49])). Furthermore, the compound AMTAC-19 exhibited interactions like those established by the DXR compounds and the PDB ligand at the residues Ile32, Val158, Leu168, Ala53, Met108, Val40, and Lys55 (hydrophobic interactions) and Met108 (steric interactions) (Figure 4).

**Figure 5:**
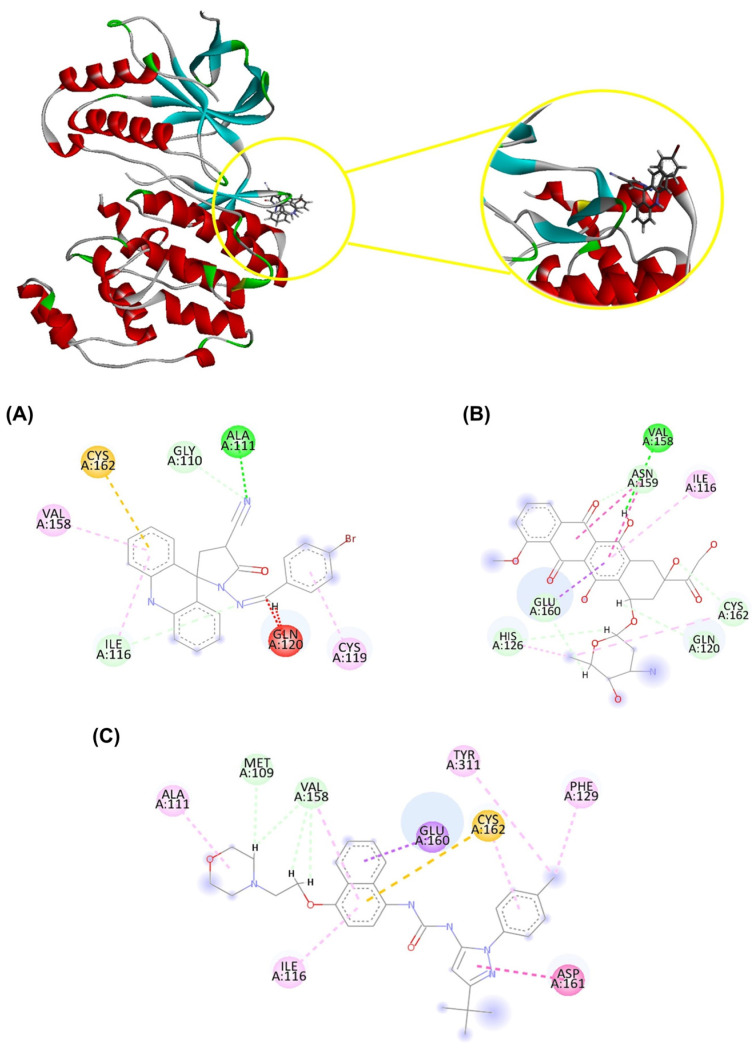
2D and 3D interactions between (**A**) AMTAC-19, (**B**) doxorubicin (DXR), (**C**) doramapimod (BIRB-796) and p38α MAPK (PDB: 1R39). Legend: pink and blue: hydrophobic interactions; red and orange: steric interactions; green: hydrogen bond interactions; HIS (Histidine); TYR (Tyrosine); PHE (Phenylalanine); ASN: asparagine; VAL: valine; ILE: isoleucine; MET: methionine; CYS: cysteine; GLY: glycine; ALA: alanine; ASP: aspartic acid; GLU: glutamic acid; and GLN: glutamine.

The interaction between p38α MAPK and the compound AMTAC-19 occurred through hydrophobic interactions corresponding to the alkyl and π-alkyl types, present in the ATP (Adenosine Triphosphate) binding domain. As observed for ERK1 and JNK1, these interactions were predominantly found in the aromatic rings of the structure through the residues Val158 (1 interaction), Ile116 (1 interaction), and Cys119 (1 interaction). Steric interactions were of the unfavorable type (dashed lines in red) with the residue Gln120 (2 interactions). Additionally, hydrogen bonds were observed between AMTAC-19 and the residues Ala111 (1 interaction), Gly110 (1 interaction), and Ile116 (1 interaction). The hydrophobic interaction formed by the Ile116 residue was observed in all compounds (Figure 5).

### 2.2. AMTAC-19 Treatment Activates the ERK1/2 and JNK1 Proteins

**Figure 6:**
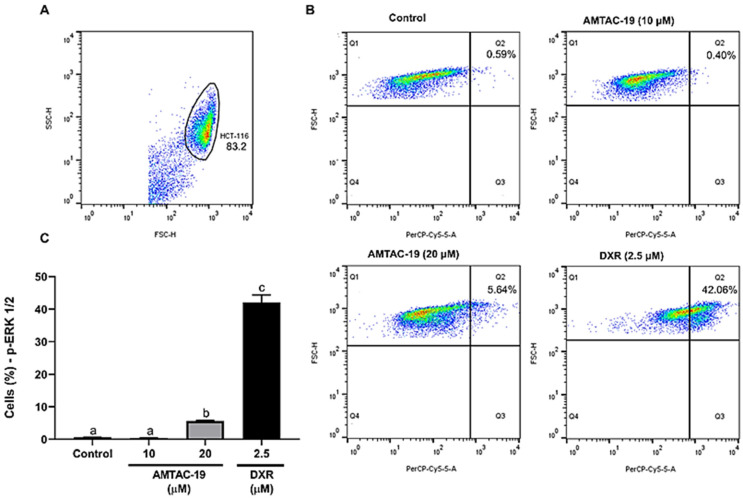
Effect of AMTAC-19 (10 or 20 µM) or doxorubicin (DXR, 2.5 µM) treatments on phospho-Extracellular Signal-Regulated Kinase 1 and 2 (p-ERK1/2) activity in HCT-116 cells. (**A**) The cell population was assessed by analyzing the p-ERK1/2 fluorescence dotplots (PerCP-Cy5.5), determining the region (Q1) corresponding to the autofluorescence of unlabeled cells and the region (Q2) corresponding to the percentage of fluorescent cells considered positive. (**B**) Representative dotplots show p-ERK1/2 fluorescence (PerCP-Cy5.5, X-axis) as a function of cell size (FSC–Forward Scatter, Y-axis) for different experimental groups. (**C**) The results obtained by flow cytometry are graphically presented. The data are represented as mean ± standard error of the mean (SEM) in triplicate. ANOVA followed by Tukey’s test. Different letters (a, b, c) indicate significant differences between groups (*p* < 0.05).

After the treatment of HCT-116 cells with AMTAC-19 (20 µM), a significant increase in the percentage of cells labeled with anti-p-ERK1/2 antibodies (5.64 ± 0.20%; *p* < 0.05) compared to the control (0.59 ± 0.06%) was observed. Additionally, the standard drug DXR also significantly increased the percentage of labeled cells (42.07 ± 2.32%, *p* < 0.05) compared to the control (Figure 6).

**Figure 7:**
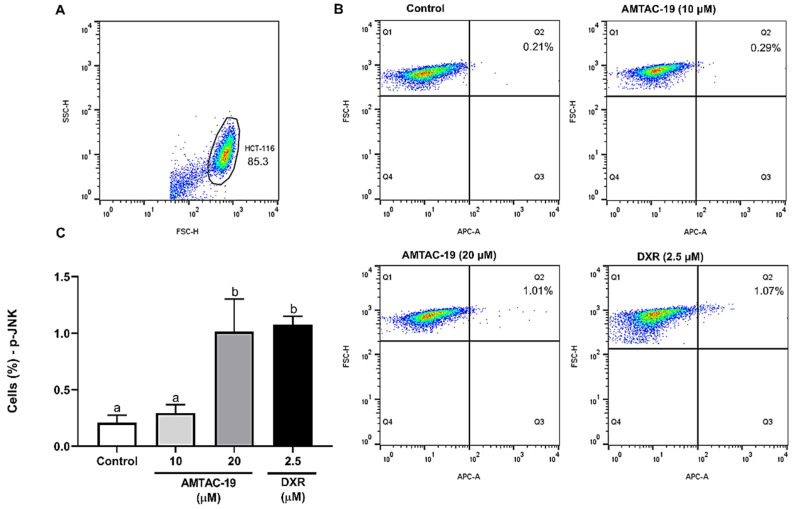
Effect of AMTAC-19 (10 or 20 µM) or doxorubicin (DXR, 2.5 µM) treatments on phospho-c-Jun N-terminal Kinase (p-JNK) activity in HCT-116 cells. (**A**) The cell population was assessed by analyzing p-JNK fluorescence dotplots (APC), determining the region (Q1) corresponding to the autofluorescence of unlabeled cells and the region (Q2) corresponding to the percentage of fluorescent cells considered positive. (**B**) Representative dotplots show p-JNK fluorescence (APC, X-axis) as a function of cell size (FSC–Forward Scatter, Y-axis) for different experimental groups. (**C**) The results obtained by flow cytometry are graphically presented. The data are represented as mean ± standard error of the mean (SEM) in triplicate. ANOVA followed by Tukey’s test. Different letters (a, b) indicate significant differences between groups (*p* < 0.05).

After 48 h of AMTAC-19 treatment (20 µM), the percentage of cells labeled with the anti-p-JNK antibody increased (1.02 ± 0.28%; *p* < 0.05) compared to the control (0.21 ± 0.06%). The standard drug DXR (2.5 µM) also induced an increased the percentage of labeled cells (1.07 ± 0.07%, *p* < 0.05) compared to the control (Figure 7).

**Figure 8:**
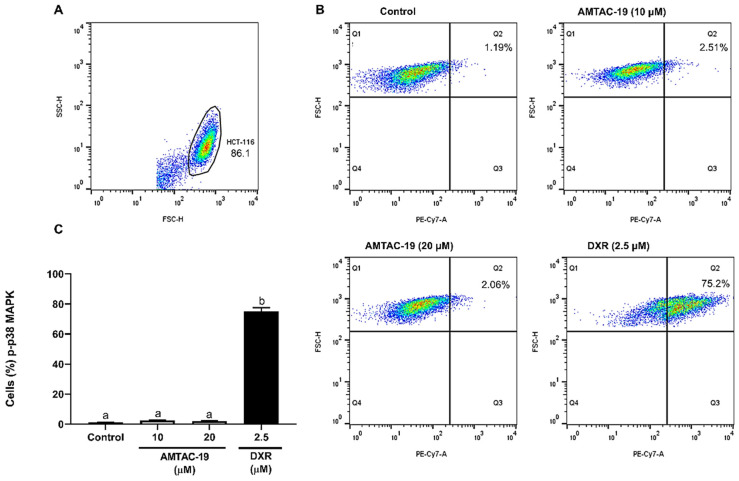
Effect of AMTAC-19 (10 or 20 µM) or doxorubicin (DXR, 2.5 µM) treatments on phospho-p38 Mitogen-Activated Protein Kinase (p-p38 MAPK) activity in HCT-116 cells. (**A**) The cell population was assessed by analyzing p-p38 MAPK fluorescence dotplots (PE-Cy7), determining the region (Q1) corresponding to the autofluorescence of unlabeled cells and the region (Q2) corresponding to the percentage of fluorescent cells considered positive. (**B**) Representative dotplots show p-p38 MAPK fluorescence (PE-Cy7, X-axis) as a function of cell size (FSC–Forward Scatter, Y-axis) for different experimental groups. (**C**) The results obtained by flow cytometry are graphically presented. The data are represented as mean ± standard error of the mean (SEM) in triplicate. ANOVA followed by Tukey’s test. Different letters (a, b) indicate significant differences between groups (*p* < 0.05).

No significant changes were observed in the percentage of cells labeled with anti-p-p38 MAPK antibodies after AMTAC-19 treatment. In contrast, DXR treatment induced a significant increase in the percentage of cells labeled with anti-p-p38 MAPK antibodies (75.20 ± 2.37%, *p* < 0.05) compared to the control (1.20 ± 0.11%, *p* < 0.05) (Figure 8).

### 2.3. The Activation of ERK and JNK Is Involved in the Cytotoxicity Induced by AMTAC-19

**Figure 9:**
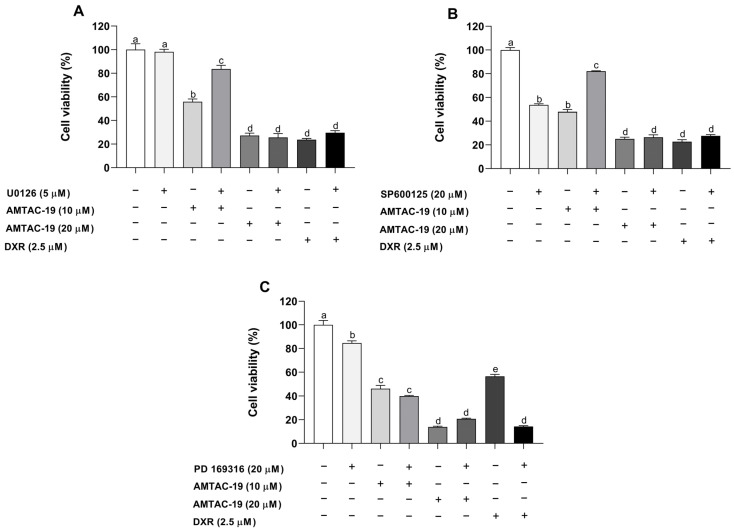
The MTT assay was used to evaluate cell viability after treatment with AMTAC-19 (10 and 20 μM) or doxorubicin (DXR, 2.5 µM) for 72 h, in the presence or absence of MAPK inhibitors: (**A**) ERK1/2 inhibitor (U0126, 5 μM), (**B**) JNK inhibitor (SP600125, 20 μM), or (**C**) p38 MAPK inhibitor (PD 169316, 20 μM). The results are presented as mean ± standard error of the mean (SEM) in triplicate. ANOVA followed by Tukey’s test. Different letters (a, b, c, d, e) indicate significant differences between groups (*p* < 0.05).

AMTAC-19 (10 μM) treatment in the absence of the ERK inhibitor (U0126; 5 μM) induced a significant reduction in cell viability (55.84 ± 2.38%, *p* < 0.05) compared to the control (100.00 ± 5.04%). ERK inhibitor (U0126, 5 μM) pretreatment significantly prevented AMTAC-19 cytotoxicity (83.67 ± 3.17%, *p* < 0.05) compared to the group treated with AMTAC-19 in the absence of the U0126 inhibitor (Figure 9A).

Similarly, AMTAC-19 (10 μM) treatment in the absence of the JNK inhibitor (SP600125, 20 μM) resulted in a significant reduction in cell viability (47.89 ± 1.98%, *p* < 0.05) compared to the control (100.00 ± 2.08%). SP600125 inhibitor pretreatment significantly prevented AMTAC-19 cytotoxicity (82.11 ± 0.48%, *p* < 0.05) compared to the group treated with AMTAC-19 in the absence of the JNK inhibitor (Figure 9B).

On the other hand, no significant effects on AMTAC-19 cytotoxicity (39.95 ± 0.61%, *p* < 0.05) were observed after pretreatment with the p38 MAPK inhibitor (PD 169316, 20 μM) compared to the group treated with AMTAC-19 (10 μM) in the absence of the PD169316 inhibitor (46.26 ± 2.79%, *p* < 0.05) (Figure 9C).

Furthermore, as observed in Figure 8C, p38 MAPK inhibitor pretreatment significantly enhanced the DXR cytotoxicity (14.74 ± 0.75%, *p* < 0.05) compared to the group treated with DXR in the absence of PD169316 inhibitor (56.49 ± 1.62%) (Figure 9C).

### 2.4. AMTAC-19 Induces Oxidative Stress in HCT-116 Cells

**Figure 10:**
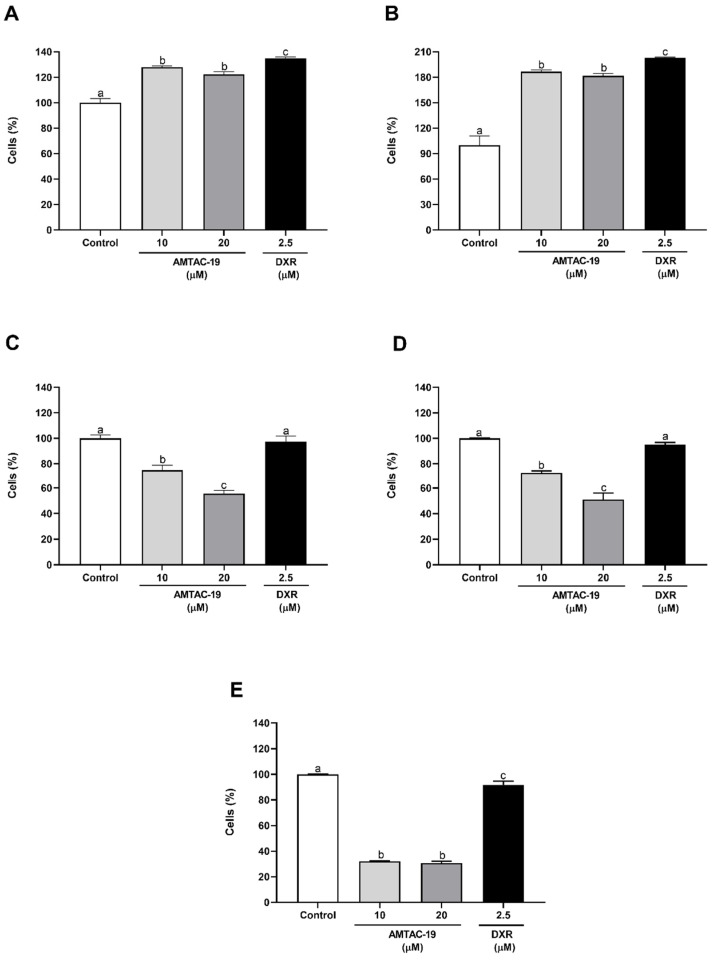
Measurement of ROS generation in HCT-116 cells treated with AMTAC-19 (10 and 20 µM) and doxorubicin (DXR) (2.5 µM) after (**A**) 30 min, (**B**) 1 h, (**C**) 6 h, (**D**) 12 h, and (**E**) 24 h of treatment with AMTAC-19 (10 and 20 μM) or doxorubicin (DXR) (2.5 µM). The results are presented as mean ± standard error of the mean (SEM) in triplicate. ANOVA followed by Tukey’s test. Different letters (a, b, c) indicate significant differences between groups (*p* < 0.05).

AMTAC-19 treatment induced a significant increase in the percentage of fluorescent cells after 30 min (10 μM: 127.70 ± 1.12%; 20 μM: 122.15 ± 2.21%, *p* < 0.05 for both) and 1 h (10 μM: 186.65 ± 1.91%; 20 μM: 181.59 ± 3.11%, *p* < 0.05 for both) compared to the control (30 min: 100.00 ± 3.22%; 1 h: 100.00 ± 10.92%) (Figure 10A,B). In contrast, AMTAC-19 treatment induced a significant reduction in the percentage of fluorescent cells after 6 h (10 μM: 74.87 ± 3.92%; 20 μM: 55.49 ± 2.54%, *p* < 0.05 for both), 12 h (10 μM: 72.73 ± 1.70%; 20 μM: 51.13 ± 4.95%, *p* < 0.05 for both), and 24 h (10 μM: 32.11 ± 0.42%; 20 μM: 30.56 ± 1.61%, *p* < 0.05 for both) compared to the control (6 h: 100.00 ± 2.62%; 12 h: 100.00 ± 0.30%, and 24 h: 100.00 ± 0.21%, *p* < 0.05 for all) (Figure 10C–E).

The DXR drug, used as a positive control, induced a significant increase in the percentage of fluorescent cells after 30 min (134.98 ± 0.84%, *p* < 0.05) and 1 h (202.76 ± 0.93%, *p* < 0.05) of treatment compared to the control (Figure 9A,B). Furthermore, a significant reduction in the percentage of fluorescent cells was observed after 24 h of treatment with this drug (91.65 ± 2.97%, *p* < 0.05) compared to the control (Figure 10E).

### 2.5. AMTAC-19 Induces ROS-Dependent Cytotoxicity in HCT-116 Cells

**Figure 11:**
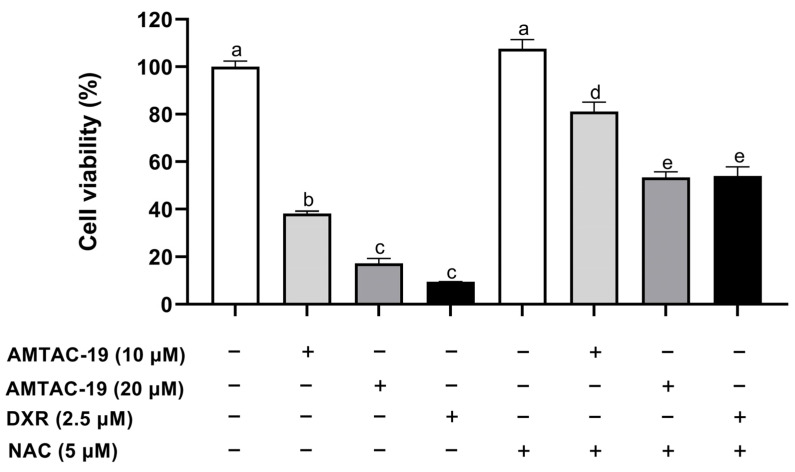
The MTT assay was used to evaluate cell viability after treatment with AMTAC-19 (10 or 20 µM) or doxorubicin (DXR) (2.5 µM) in the presence or absence of N-acetylcysteine (NAC, 5 µM) for 72 h. The results are presented as mean ± standard error of the mean (SEM) in triplicate. ANOVA followed by Tukey’s test. Different letters (a, b, c, d, e) indicate significant differences between groups (*p* < 0.05).

As shown in Figure 11, AMTAC-19 treatment (10 and 20 μM) in the absence of N-acetylcysteine (NAC, 5 μM) significantly reduced the cell viability (10 μM: 38.21 ± 1.04%; 20 μM: 17.29 ± 2.06%, *p* < 0.05 for both) compared to the control (100.00 ± 2.48%). NAC pretreatment significantly prevented the AMTAC-19 cytotoxic effect (cell viability—10 μM: 81.14 ± 3.96%; 20 μM: 53.40 ± 2.48%, *p* < 0.05 for both) compared to the groups treated with AMTAC-19 (10 and 20 μM) in the absence of NAC.

Additionally, NAC pretreatment induced a significant reduction in cytotoxicity of the standard drug DXR (9.42 ± 0.17%, *p* < 0.05) compared to the group treated with DXR in the absence of NAC (54.09 ± 3.75%) (Figure 11).

## 3. Discussion

Colorectal cancer (CRC) has a significant global incidence and is responsible for high cancer mortality rates [50]. As a result, the search for new therapeutic alternatives remains relentless [50,51]. In this context, spiro-acridine compounds have emerged as promising candidates, with these molecules being extensively investigated for their potential in cancer therapy [46,47]. The spiro-acridine derivative (*E*)-1′-((4-bromobenzylidene)amino)-5′-oxo-1′,5′-dihydro-10*H*-spiro[acridine-9,2′-pyrrole]-4′-carbonitrile (AMTAC-19) was previously synthesized and demonstrated significant cytotoxicity in the HCT-116 colorectal cancer cell line (half maximal inhibitory concentration, IC50 = 10.35 ± 1.66 µM) through cellular redox state alteration and apoptosis induction [48]. To better characterize the anti-CRC effect of AMTAC-19, we investigated the molecular docking between AMTAC-19 and the crystallographic structures of Mitogen-Activated Protein Kinases (MAPKs). In addition, in vitro assays using the HCT-116 cell line were performed to evaluate the effect of AMTAC-19 on MAPKs modulation and Reactive Oxygen Species (ROS) production.

Apoptosis induction is one of the main antitumor mechanisms by which acridine derivatives act [4,11,12,52,53]. Overactivation of Extracellular Signal-Regulated Kinase 1 and 2 (ERK1/2) and c-Jun N-terminal Kinase (JNK) is observed in CRC [54]. However, the dual role of ERK1/2 and JNK in various types of cancer has been widely reported [55]. Thus, the stimulation of these kinases’ activity may be involved in either pro-tumor or anti-tumor effects, including in CRC [56,57]. The involvement of MAPKs in the induction of tumor cell death has been reported, which is dependent on the cell type and the stimulus [58]. In CRC cells, the activation of ERK1/2 and JNK is associated with the activation of apoptosis [29,59,60,61]. Currently, various stimuli have been linked to this tumor cell death-inducing effect mediated by the activation of ERK1/2 and JNK, which includes the generation of Reactive Oxygen Species (ROS) [62,63,64]. The results of molecular docking showed a favorable interaction between AMTAC-19 and the ERK1 through amino acid residues that are also involved in the interaction between this protein and the Protein Data Bank (PDB) ligand or with the standard drug DXR (Leu156, Ala52, Ile31, Thr110, and Asp111). Additionally, AMTAC-19 demonstrated interaction with the active site of ERK1 through Cys166, which is a crucial residue for the inhibition mechanism and regulation of ERK activity [34]. As reported by Gao et al. (2022) [31], buxifoliadine E, an acridine compound, can interact with the catalytic segment of the ERK protein through hydrogen bonds between the Lys54 and Glu71 residues and by interacting with Asp167 in the KDD motif of the catalytic segment of this enzyme. In addition, Boshta et al. (2024) [65] synthesized 1,3,5-trisubstituted-1H-pyrazole derivatives, namely compounds 6, 7, 10a, 10c, and 10d, which exhibited significant cytotoxicity against prostate (PC-3) and breast (MCF-7) cancer cell lines. In the molecular docking analysis, it was observed that compounds 6, 10a, and 10d, the most cytotoxic in the in vitro assays, were able to interact with the ERK protein through the Ala52, Thr110, and Asp111 residues.

AMTAC-19 also exhibited favorable interactions with the JNK1 protein through hydrophobic interactions between the amino acid residues Ile32, Val158, Leu168, Ala53, Met108, Val40, and Lys55. Additionally, the compound exhibited steric interactions through residue Met108, which are essential for the enzyme’s activity and responsible for controlling catalytic function [66,67]. Malki et al. (2015) [68] obtained new thiosemicarbazides and 1,3,4-oxadiazoles and evaluated their cytotoxic effects against the MCF-7 cell line. Among the tested compounds, 2-(3-(4-chlorophenyl)-3-hydroxybutanoyl)-N-phenylhydrazinocarbonothioamide, designated as 4c, exhibited the highest cytotoxicity and, after molecular docking analysis, showed favorable interactions with residues Ile32, Leu168, and Val158 of JNK.

The favorable interactions observed between the p38α MAPK protein and AMTAC-19 involved amino acid residues associated with the catalytic site of this enzyme (hydrophobic interactions: Val158, Ile116, and Cys119; hydrogen bonds: Ala111, Gly110, and Ile116; and steric interactions: Gln120, Cys119, and Cys162) [69,70]. Similarly, the residue Ile116 is involved in the interaction between p38α MAPK and the ligand doramapimod (BIRB-796), a selective inhibitor of this protein. Kaboli et al. (2019) evaluated the interaction between berberine, an alkaloid compound structurally similar to DNA intercalating agents, and p38α MAPK. These authors observed that berberine interacts with p38α MAPK through Gly110, which is also involved in the binding of AMTAC-19 and this enzyme [71].

Our molecular docking results suggest that the investigated proteins are potential molecular targets for the anti-CRC effect of AMTAC-19. In this regard, we conducted in vitro tests to confirm the involvement of ERK, JNK, and p38 MAPK in the antitumor activity of this spiro-acridine compound.

The labeling of HCT-116 cells with anti-p-ERK1/2 and anti-p-JNK1 antibodies reveals that treatment with AMTAC-19 induces the activation of these enzymes. Supporting the role of ERK1/2 and JNK1 in AMTAC-19 cytotoxicity, pretreatment with ERK1/2 (U0126) and JNK (SP600125) inhibitors partially prevented the anti-proliferative effect of AMTAC-19. This finding corroborates the antitumor effect of the acridine benzimidazole derivative N-{(1H-benzo[d]imidazol-2-yl)methyl}-2-butylacridin-9-amine (8m), which induced JNK-mediated apoptosis in HCT-116 cells [72]. Similarly, brucein D, a quassinoid compound, induces apoptosis and autophagy in lung cancer cells (A549 and NCI-H292), mediated by ROS generation and activation of ERK and JNK [73].

On the other hand, the use of anti-p-p38 MAPK antibodies and pretreatment with the p38 MAPK inhibitor (PD 169316) revealed that there is no involvement of p38 MAPK in the anti-CRC effect of AMTAC-19.

Therefore, our results demonstrate that the activation of ERK1/2 and JNK1 proteins is involved in the in vitro antitumor effect induced by AMTAC-19.

Various signals can modulate MAPK activity, including ROS [74]. Our previous data demonstrated that AMTAC-19 reduces ROS production after 48 h of treatment. However, given that antineoplastic compounds can induce increased ROS levels in the early stages of treatment and activate MAPKs signaling pathways [62], we suggest that the modulation of the redox state in HCT-116 cells, shortly after AMTAC-19 treatment, could be linked to oxidative stress induction and subsequent MAPKs activation. Therefore, we decided to evaluate ROS levels at time points earlier than 48 h (30 min, 1 h, 6 h, 12 h, and 24 h) using the 2,7-dichlorodihydrofluorescein diacetate (DCFH-DA) assay. Elevated ROS levels were observed after 30 min and 1 h of AMTAC-19 treatment, followed by a progressive reduction in ROS levels after this period. Supporting the evidence that the anti-CRC effect of AMTAC-19 depends on the initial ROS production, pretreatment with N-acetylcysteine (NAC), an antioxidant compound [75], significantly prevented the cytotoxicity of this molecule. Chen et al. (2015) [72] reported that a novel benzimidazole acridine derivative, designated as 77, induced the death of HCT-116 cells through the induction of ROS production. Thus, we suggest that the induction of oxidative stress is involved in ERK and JNK activation after AMTAC-19 treatment in HCT-116 cells.

## 4. Materials and Methods

### 4.1. Drugs and Reagents

BD Phosflow™ Alexa Fluor^®^ 647 mouse anti-p-JNK (pT183/pY185) (BD Biosciences^®^, Franklin Lakes, NJ, USA), BD Phosflow™ PE-Cy™7 mouse anti-p-p38 MAPK (pT180/pY182) (BD Biosciences^®^, NJ, USA), BD Phosflow™ PerCP-Cy™5.5 mouse anti-p-ERK1/2 (pT202/pY204) (BD Biosciences^®^, NJ, USA), Buffered phosphate solution (PBS) (Sigma-Aldrich^®^; St. Louis, MO, USA), dimethylsulfoxide (DMSO) (Dinâmica^®^, Indaiatuba, SP, Brazil), doxorubicin (DXR) (Sigma-Aldrich^®^, St. Louis, MO, USA), Fetal Bovine Serum (FBS) (GIBCO^®^, Grand Island, NY, USA), 3-(4,5-dimethylthiazol-2-yl)-2,5-diphenyltetrazolium bromide (MTT) (Sigma-Aldrich^®^, St. Louis, MO, USA), penicillin–streptomycin (Sigma-Aldrich^®^, St. Louis, MO, USA), PD 169316 (Sigma-Aldrich^®^, St. Louis, MO, USA), Roswell Park Memorial Institute 1640 (RPMI) (Sigma-Aldrich^®^, St. Louis, MO, USA), Sodium Dodecyl Sulfate (SDS) (Êxodo Científica^®^, Sumaré, SP, Brazil), SP600125 (Sigma-Aldrich^®^, St. Louis, MO, USA), trypsin 0.25% with ethylenediaminetetraacetic acid (GIBCO^®^, Grand Island, NE, USA), U0126 (Sigma-Aldrich^®^, St. Louis, MO, USA).

The drugs and reagent solutions were prepared immediately before use.

### 4.2. Chemistry

AMTAC-19 was synthesized at the Drug Development and Synthesis Laboratory (LDSF) of the State University of Paraíba (UEPB) (Campina Grande, PB, Brazil) by Prof. Dr. Ricardo Olimpio de Moura, as previously described [3].

### 4.3. HCT-116 Colorectal Carcinoma Cell Line

The HCT-116 human tumor cell line was obtained from the Rio de Janeiro Cell Bank (BCRJ), Duque de Caxias, RJ, Brazil. Cells were maintained in RPMI medium, supplemented with 10% Fetal Bovine Serum and 1% penicillin–streptomycin, and incubated at 37 °C in a 5% CO_2_ atmosphere.

### 4.4. Docking Prediction

The molecular docking simulation was used to predict the interaction between the compound AMTAC-19 and the following proteins: Extracellular Signal-Regulated Kinase 1/2 (ERK 1/2) (Protein Data Bank (PDB): 5LCJ) in complex with the pyridine carbamate inhibitor, with a resolution of 1.78 Å [56,76]; c-Jun N-terminal Kinase 1 (JNK1) (PDB: 2G01) in complex with the pyrazoloquinolone inhibitor, with a resolution of 3.50 Å [49]; and p38 Mitogen-Activated Protein Kinase α (p38α MAPK) (PDB ID: 1R39) with a resolution of 2.30 Å [69], obtained via X-ray diffraction. Doramapimod (BIRB-796) was used as the control ligand for the p38α MAPK protein [77]. The three-dimensional (3D) structures of the proteins studied were obtained from the PDB (https://www.rcsb.org/pdb/home/home.do—accessed on 24 November 2023).

Prior to the start of the computational simulation, the chemical structures of the compounds were designed using Marvin Sketch v.23.10, ChemAxon (https://chemaxon.com/marvin, accessed on 1 July 2024), followed by the standardization of the chemical structures in 3D and the energy minimization of the compounds using molecular mechanics methods and the semi-empirical Austin Model 1 (AM1) method, through Spartan 14 software, WaveFunction (https://www.wavefun.com/). All water molecules were removed from the crystalline structure, and a template between the enzyme and the co-crystallized ligand was created to mark the active site of the macromolecule. Next, the test molecule was inserted into the enzyme’s active site, and the molecular docking simulation was performed.

The re-docking technique was applied, therefore the Root Mean Square Deviation (RMSD) from docking poses of the co-crystallized ligand was calculated, indicating the degree of reliability of the fit. The RMSD provides the connection mode close to the experimental structure and is considered successful if the value is below 2.0 Å.

For the p38α MAPK (PDB ID: 1R39) mechanism, which did not have a co-crystallized ligand, the active site was identified by visualizing the residues corresponding to the active site from the PDB-related articles, as well as using molecular pocket predictions. The Bite Net—Skolteck I Molecule platform (https://sites.skoltech.ru/imolecule/tools/bitenet—accessed on 1 July 2024) was used. For the p38α MAPK protein (PDB ID: 1R39), the active site coordinates were X = 55.93, Y = 33.48, and Z = 39.84.

Molegro Virtual Docker v.6.0.1 (MVD) software was used with predefined parameters. The co-crystallized ligand was used to define the active site. The compounds were then inserted to analyze the system’s stability through the interactions identified with the enzymes’ active sites, referencing the MolDock Score energy value. The MolDock SE (Simplex Evolution) algorithm was employed with the following parameters: A total of 30 runs with a maximum of 3000 interactions using a population of 50 individuals, 2000 minimization steps for each flexible residue, and 2000 global minimization steps per simulation. The MolDock Score (GRID) scoring function was used to calculate docking energy values. A GRID was set at 0.3 Å, and the search sphere was fixed at a radius of 15 Å. For the analysis of ligand energy, internal electrostatic interactions, internal hydrogen bonds, and sp2-sp2 torsions were evaluated [78,79].

### 4.5. Assessment of MAPK Activity After AMTAC-19 Treatment Using Flow Cytometry

In this experiment, cell culture was performed using HCT-116 cells (1 × 10^6^ cells/mL) in 24-well plates with the compound AMTAC-19 at concentrations of 10 and 20 µM for 48 h. Doxorubicin (DXR) at 2.5 µM was used as the standard drug. Following the treatment period, the cells were detached from the plate using a trypsin/EDTA solution and centrifuged (500× *g*, 5 min, 20 °C), then resuspended in buffer and fixed for 10 min at 37 °C. The treated samples were washed, permeabilized with lysis buffer, and resuspended with anti-p-ERK1/2, anti-p-JNK, or anti-p-p38 MAPK antibodies, and incubated for 30 min at 25 °C in the dark. Subsequently, the cells were washed, centrifuged (300× *g*, 5 min, 4 °C), and the supernatant was removed according to the manufacturer’s instructions. The cells were then resuspended in buffer and analyzed by flow cytometry (10,000 events per sample). The experiments were independently conducted in triplicate, and the obtained data were analyzed using DIVA 6.0 software [56].

### 4.6. Evaluation of AMTAC-19 Cytotoxicity in the Presence or Absence of MAPKs Inhibitors

The MTT assay was utilized to evaluate the cytotoxicity induced by AMTAC-19. HCT-116 cells (3 × 10^5^ cells/mL) were cultured in 96-well plates and treated in the presence or absence of MAPK inhibitors: 5 μM of the ERK inhibitor (U0126), 20 μM of the JNK inhibitor (SP600125), or 20 μM of the p38 MAPK inhibitor (PD 169316) for 3 h. Following treatment, the cells were centrifuged, and the supernatant was removed, after which 10 µL of MTT solution (5 mg/mL) was added to each well and incubated for 4 h in 5% CO_2_ at 37 °C. Subsequently, SDS (100 μL per well) was added to dissolve the formed formazan crystals. The analysis was performed by measuring the optical density using a microplate reader (Synergy HT, BioTek^®^, Winooski, VT, USA) at 570 nm. Three independent experiments were conducted in duplicate [56].

### 4.7. Quantification of Reactive Oxygen Species (ROS) by the DCFH-DA Assay

2,7-dichlorodihydrofluorescein diacetate (DCFH−DA) is a fluorogenic reagent that was used to assess Reactive Oxygen Species (ROS) in HCT-116 cells following AMTAC-19 treatment. HCT-116 cells were seeded in 24-well plates (1 × 10^6^ cells/mL) and incubated for 24 h (5% CO_2_ at 37 °C). Then, the cells were treated with AMTAC-19 (10 or 20 µM), DXR (2.5 µM), or hydrogen peroxide (H_2_O_2_) (500 µM) in the presence of DCFH−DA (10 µM) and incubated for 30 min, 1 h, 6 h, 12 h, or 24 h in 5% CO_2_ at 37 °C. After this period, the cells were collected and resuspended in PBS. The percentage of fluorescent cells was determined using flow cytometry, analyzing 10,000 events acquired at excitation wavelengths of 485 nm and fluorescence wavelengths of 530 nm. Three independent experiments were conducted in duplicate [80].

### 4.8. Assessment of Oxidative Stress Involvement in AMTAC-19 Cytotoxicity

The assessment of ROS involvement in AMTAC-19 cytotoxicity was performed using the MTT assay in the presence or absence of N-acetylcysteine (NAC), an antioxidant compound. HCT-116 cells were seeded in 96-well plates (100 µL/well) at a density of 3 × 10^5^ cells/mL and incubated for 24 h (37 °C, 5% CO_2_). After this period, the cells were incubated for an additional 3 h (37 °C, 5% CO_2_) in the presence or absence of 5 µM NAC. The cells were then treated with AMTAC-19 (10 or 20 µM, 100 µL per well) or DXR (2.5 µM, 100 µL per well) and incubated for 72 h in a 5% CO_2_ atmosphere at 37 °C. Following incubation, the plates were centrifuged, and 110 µL of the supernatant was removed. Subsequently, MTT solution (5 mg/mL, 10 µL per well) was added, and the cells were incubated for 4 h at 37 °C in 5% CO_2_. The formazan crystals were dissolved in 100 µL of SDS, and the optical densities were measured using a microplate reader (Synergy HT, BioTek^®^) at a wavelength of 570 nm. Three independent experiments were conducted in triplicate [80].

### 4.9. Statistical Analysis

Statistical analyses were conducted using version 8.0.2 of GraphPad Prism (GraphPad Software Inc., San Diego, CA, USA). The results are presented as the mean ± standard error of the mean (SEM). Data were subjected to one-way Analysis of Variance (ANOVA), followed by Tukey’s test (*p* < 0.05).

## 5. Conclusions

Our data corroborated the significant in vitro antitumor effect of AMTAC-19 in the HCT-116 cell line (human colorectal cancer, CRC). It is important to highlight that Reactive Oxygen Species (ROS) have the capacity to activate different cellular signaling pathways, including Extracellular Signal-Regulated Kinases 1 and 2 (ERK1/2) and c-Jun N-terminal Kinase (JNK). The activation of these enzymes is related to crucial cellular processes, including the apoptosis induction in tumor cells. Based on this interaction between ROS and MAPKs, we proposed that the initial redox changes induced by AMTAC-19 treatment play a fundamental role in the ERK1/2 and JNK activation. This effect may lead to subsequent apoptosis in HCT-116 cells. The implications of these findings are significant, as they suggest that AMTAC-19 not only induces oxidative stress in tumor cells but also activates signaling pathways that culminate in cell death. Considering these discoveries, our results encourage further investigations to better understand the underlying mechanisms of the anti-CRC effects of AMTAC-19, which could contribute to the development of more effective therapeutic strategies against CRC. Additionally, our results also provide insights into how ROS and MAPKs may serve as targets in cancer pharmacotherapy.

## Figures and Tables

**Table 1:** MolDock score (arbitrary units) and interaction probability values (*p*) between the (*E*)-1′-((4-bromobenzylidene)amino)-5′-oxo-1′,5′-dihydro-10*H*-spiro[acridine-9,2′-pyrrole]-4′-carbonitrile (AMTAC-19) and Extracellular Signal-Regulated Kinase 1 (ERK1), c-Jun N-terminal Kinase 1 (JNK1), and p38 Mitogen-Activated Protein Kinase α (p38α MAPK).

Protein	Ligand	MolDock Score *	(*p*) ** MolDock Score	Critical Residues	Distance of Hydrogen Bonding Interactions (Å)
ERK1	AMTAC-19	−84.735	0.793	Leu156, Ala52, Ile31 and Thr110 (hydrophobic interactions); Asp111 (hydrogen bond)	Val 39 (2.86 Å).
	Doxorubicin	−83.988	0.786		Leu 107 (2.38 Å); Met 108 (2.15 Å); Ile31 (2.50 Å); Glu 109 (2.32 Å); Lys 114 (2.80 Å).
	Pyridine carbamate inhibitor	−106.816	1		Lys 114 (2.29 Å); Met 108 (1.96 Å, 1.29 Å); Asp 106 (2.41 Å).
JNK1	AMTAC-19	−74.978	0.781	Ile32, Val158, Ala53, Met108, Val40 and Lys55 (hydrophobic interactions); Met108 (steric interactions).	-
	Doxorubicin	−96.058	1		Ile 32 (2.26 Å); Met 111 (1.89 Å, 2.95 Å); Leu 110 (2.54Å).
	Pyrazoloquinolone inhibitor	−68.224	0.710		Met 111 (2.20 Å, 2.99 Å).
p38α MAPK	AMTAC-19	−107.433	0.824	Ile116 (hydrophobic interactions)	Ile 116 (3.36 Å); Gly 110 (2.16 Å); Ala 111 (2.62 Å);
	Doxorubicin	−108.908	0.835		His 126 (2.74 Å); Glu 160 (2.37 Å), Val 158 (3.75 Å); Asn 159 (2.73 Å); Cys 162 (2.23 Å); Gln 120 (2.24 Å)
	BIRB-796 ^a^	−130.409	1		Met 109 (2.29 Å); Val 158 (2.33 Å, 2.70 Å, 2.80 Å).

^a^ BIRB-796: doramapimod; * Energy values expressed in arbitrary units; ** Affinity probability value.

## Data Availability

Data are contained within the article and Supplementary Materials.

## Supplementary Materials

The following supporting information can be downloaded at: https://www.mdpi.com/article/10.3390/molecules29225344/s1, Figure S1: Molecular structures of AMTAC-01 and its structural congeners AMTAC-06, AMTAC-17; Figure S2: Synthetic route to obtain AMTAC-19.
