# Supplementary material for: AMTAC-19, a Spiro-Acridine Compound, Induces In Vitro Antitumor Effect via the ROS-ERK/JNK Signaling Pathway
Source: Molecules. 2024 Nov 13;29(22):5344. doi: 10.3390/molecules29225344 (PMC11596224; doi:10.3390/molecules29225344)
Supplement: Supplementary file 1 [file molecules-29-05344-s001.zip › molecules-3300265-supplementary/Supplementary Materials/Supplementary Materials.pdf]

SUPPORTING INFORMATION

AMTAC-19, a Spiro-Acridine Compound, Induces in Vitro Antitumor Effect via the ROS-ERK/JNK Signaling Pathway

Valgrícia Matias de Sousa 1, Sâmia Sousa Duarte 1, Rafael Carlos Ferreira 1, Natália Ferreira de Sousa 1, Marcus Tullius Scotti 1, Luciana Scotti 1, Marcelo Sobral da Silva 1, Josean Fechine Tavares 1, Ricardo Olímpio de Moura 2, Juan Carlos Ramos Gonçalves 1 and Marianna Vieira Sobral 1,*

1 Postgraduate Program in Natural Products and Bioactive Synthetics, Federal University of Paraíba, João Pessoa 58051-970, PB, Brazil
2 Drug Development and Synthesis Laboratory, Department of Pharmacy, State University of Paraíba, João Pessoa 58070-450, PB, Brazil
* Correspondence: mariannavbs@gmail.com

CONTENT

Figure S1. Molecular structures of AMTAC-1 and its structural congeners AMTAC-06, AMTAC-17, and AMTAC-19 3

Figure S2. Synthetic route to obtain AMTAC-19 3

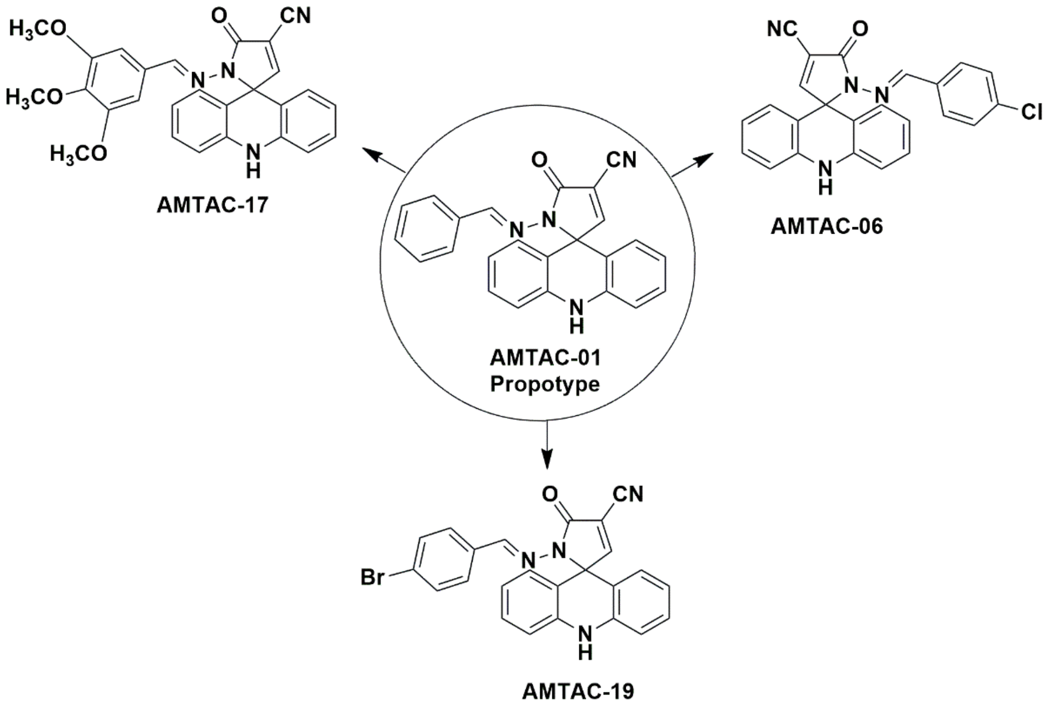

Figure S1. Molecular structures of AMTAC-1 and its structural congeners AMTAC-06, AMTAC-17, and AMTAC-19.

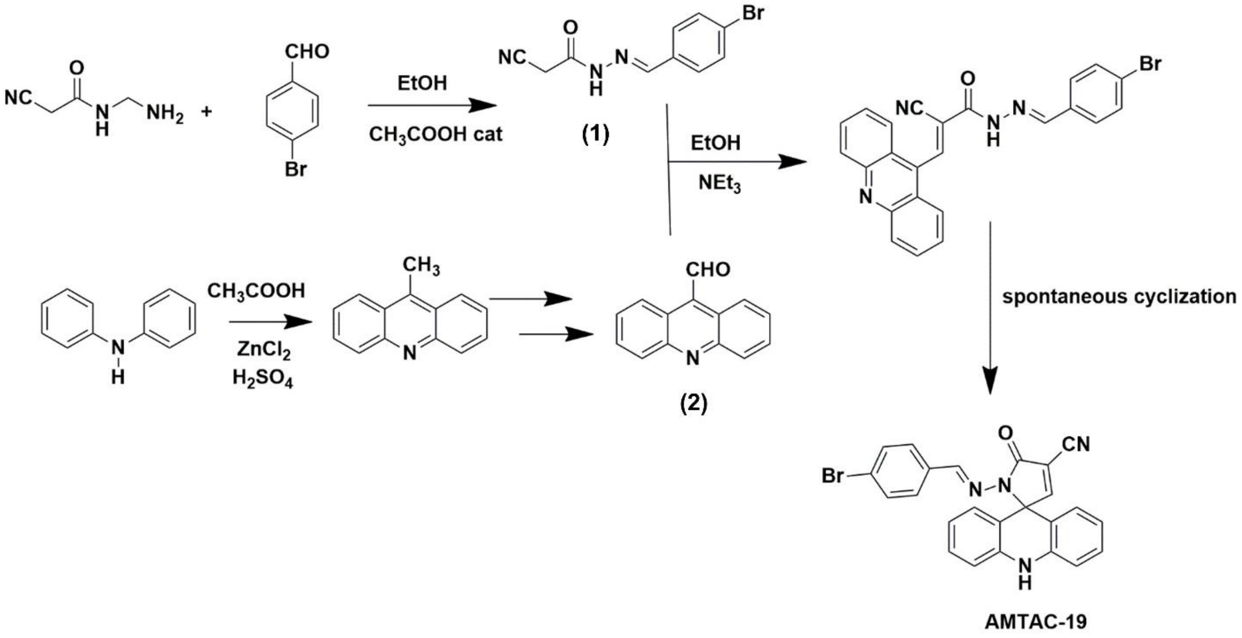

Figure S2. Synthetic route to obtain AMTAC-19. The intermediate 2-cyano-N'-(4-bromo-benzylidene)-acetohydrazide (JR-25) was used for the synthesis of AMTAC-19. JR-25 (1) was produced by the condensation of 2-cyano-acetohydrazide with 4-bromo-benzaldehyde in an acidic medium. In parallel, the acridine aldehyde (2) was synthesized from diphenylamine through Friedel–Crafts acylation, followed by cyclization and oxidation. Subsequently, the final product was obtained by the reaction between the acridine aldehyde and JR-25 in a basic medium, resulting in spontaneous cyclization.
