# Supplementary figures and images for: AMTAC-19, a Spiro-Acridine Compound, Induces In Vitro Antitumor Effect via the ROS-ERK/JNK Signaling Pathway
Source: Molecules. 2024 Nov 13;29(22):5344. doi: 10.3390/molecules29225344 (PMC11596224; doi:10.3390/molecules29225344)

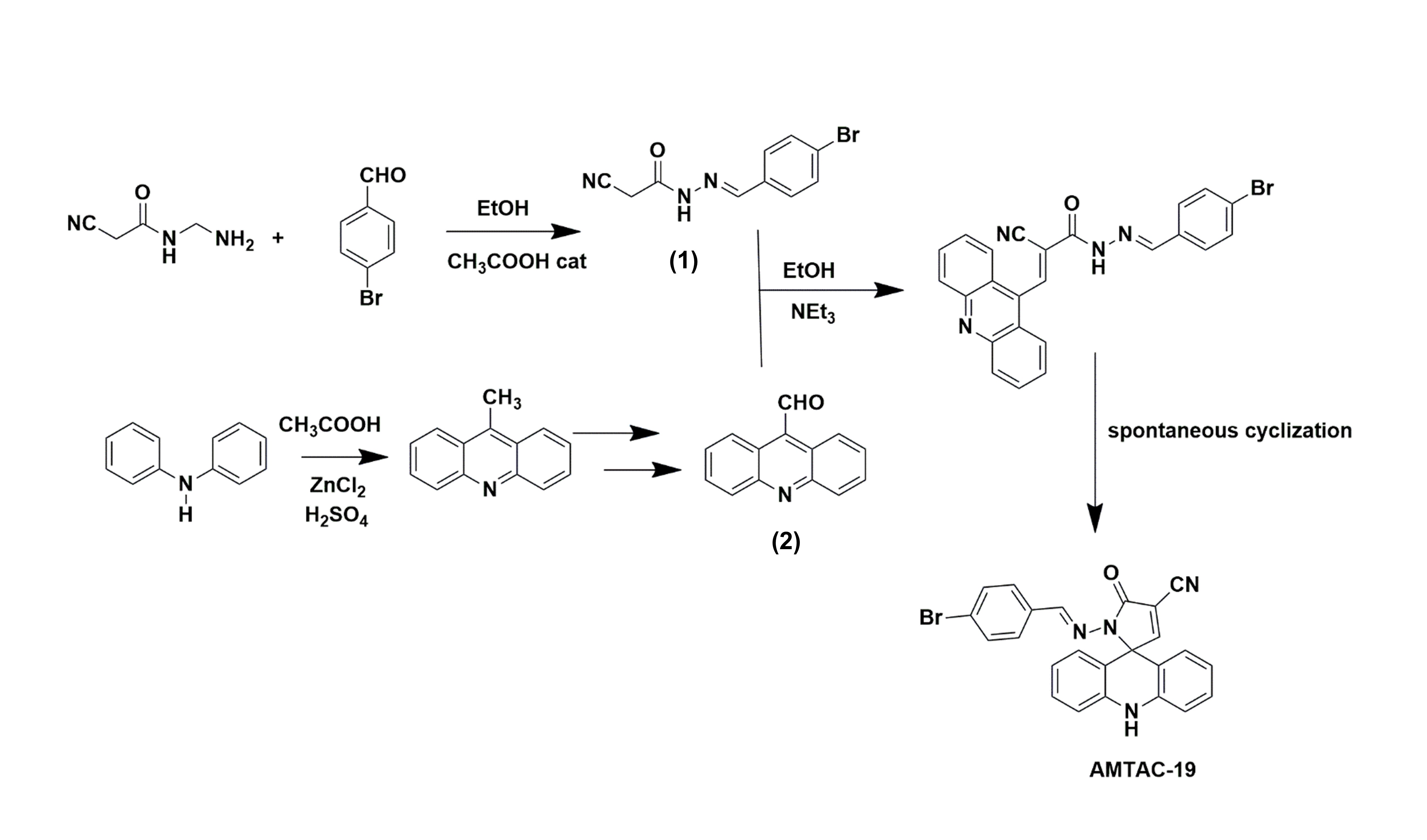

Supplement: Supplementary file 1 [file molecules-29-05344-s001.zip › molecules-3300265-supplementary/Supplementary Materials/Figure S1.jpg]

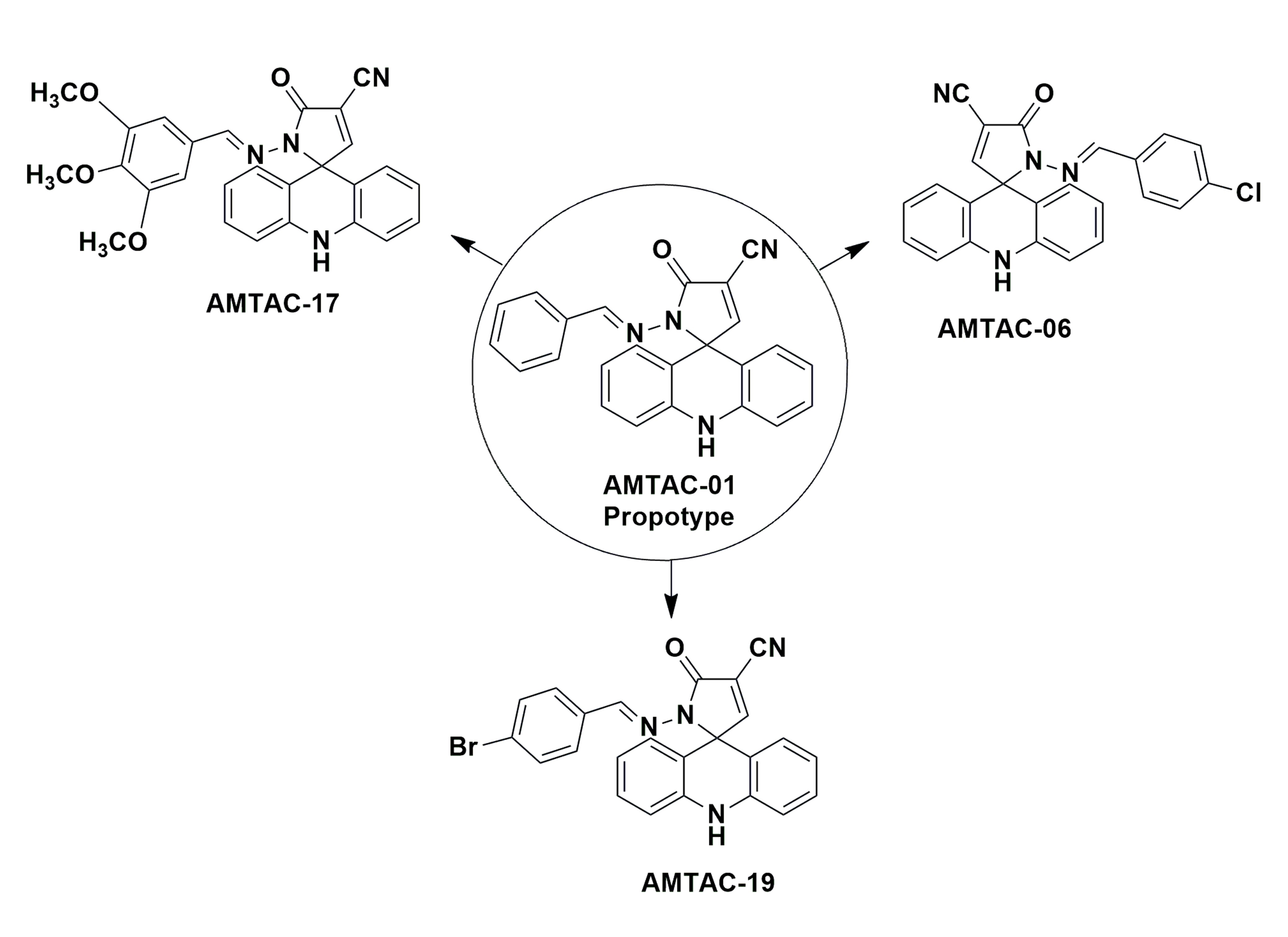

Supplement: Supplementary file 1 [file molecules-29-05344-s001.zip › molecules-3300265-supplementary/Supplementary Materials/Figure S2.jpg]
